# Research highlights for issue 6: the CRISPR/Cas revolution

**DOI:** 10.1111/eva.12279

**Published:** 2015-06-15

**Authors:** Britt Koskella

**Affiliations:** Evolutionary Applications

The evolution of host defenses against parasites and pathogens has resulted in a wide array of mechanisms conferring resistance and tolerance. Many of these adaptations have been co-opted for use in the treatment of disease, for example the use of live vaccines to prime the host immune system through the memory of B and T cells or the creation of transgenic crop plants to increase resistance to pests and pathogens (e.g., Schoonbeek et al. 2015; Tripathi et al. 2015). Indeed, the acquisition of basic knowledge regarding host–pathogen coevolution has underpinned much of the advancement in applied sciences of healthcare and disease management. Few such examples, however, have generated the widespread excitement and rapid development as the CRISPR/Cas system discovered in bacterial and archaeal genomes.

When bacteria coevolve with their bacteriophage viruses, they typically face strong selection to recognize and resist infection by circulating phage genotypes. Among the many mechanisms that have evolved in response to this pressure is the CRISPR/Cas system, which provides adaptive immunity to its host against specific phages. The system is built from clustered regularly interspaced short palindromic repeats (CRISPRs) within the genome that act together with CRISPR-associated (Cas) proteins to target and destroy foreign nucleic acids, including those from viruses and plasmids (reviewed in Barrangou 2015).

In the laboratory, experimental coevolution between bacteria and phages has been used to uncover the exact mechanisms of resistance and counter-adaptation as well as to determine the potential ecological and evolutionary impacts of such coevolution in shaping microbial populations and communities. Recent work by David Paez-Espino and coauthors has clearly demonstrated that phage populations respond rapidly to CRISPR-mediated immunity both through the accumulation of single nucleotide polymorphisms within the region of the phage genome targeted by CRISPR and via rampant recombination among phage types. Using long-term experimental coevolution of *Streptococcus thermophiles* and phage 2972, they were able to track specific evolutionary responses of the phage populations through deep sequencing and show that mutation rates were much higher than those of corresponding host populations (Paez-Espino et al. 2015). Such a rapid response by phages suggests bacterial host populations will be under a constant selection pressure to renew resistance, and emphasizes the power of the CRISPR/Cas system to confer such evolutionary flexibility.

In natural populations, bacteria–phage coevolution has also been shown to occur rapidly under CRISPR-mediated selection. Laura Sanguino and collaborators have elegantly demonstrated that CRISPR sequences obtained through metagenomics can be used to build bioinformatics networks that link viruses with their coevolving hosts (Sanguino et al. 2015). Using Arctic glacier ice and soil samples, the authors compared the direct repeats of microbial origin and short sequence spacers of viral origin that make up the CRISPR region to uncover the interaction dynamics of hosts and their, often broad host range, viruses. They found more abundant CRISPRs in ice samples relative to soil, possibly indicating higher viral diversity and infectivity rates (although they note this may also be due to limited depth of coverage in the soil metagenome dataset), and evidence for phage-mediated transduction in the bacterial community.

Now, this mechanism of prokaryotic immunity is being successfully developed as a genome-editing tool, including the engineering of mammalian cells. The CRISPR/Cas system holds the potential to knockout specific regions of the genome, alter multiple loci simultaneously, and selectively manipulate gene expression over time. This newly emerging tool not only promises to revolutionize the field of genetics, but also has direct application to the treatment of disease (reviewed in Pellagatti et al. 2015). For example, the Cas9-based DNA editing system is being exploited to help combat viral diseases through the identification of human genes linked to viral replication and the direct targeting of DNA viruses within the human body (reviewed in Kennedy & Cullen 2015). Work by Hsin-Kai Liao and colleagues recently demonstrated how the CRISPR/Cas9 system can be adapted to human cells in order to mount intracellular defense against HIV-1 infection (Liao et al. 2015). Their work shows that engineered cells expressing HIV-targeted CRISPR/Cas9 can be used both to disrupt viral DNA integrated into the host genome and to prevent new viral infection, emphasizing the great therapeutic potential of the system.

The breadth of utility for the CRISPR/Cas system is only beginning to be uncovered, with potential applications ranging from cancer screening (Chen et al. 2015) to editing of crop plant genomes (Belhaj et al. 2015). Among the many perceived benefits of this new technology is the fact that it bypasses the current GMO legislation (Kanchiswamy et al. 2015) and, unlike transgenic crop production (Tabashnik et al. 2015), allows flexible and adaptive genome editing that can be used to stay ahead of any pest and pathogen counter-adaptation. However, the ethical issues surrounding CRISPR/Cas genome editing, especially in the case of altered human embryos (Kaiser & Normile 2015), has yet to be fully addressed and the scientific community must now come together to balance the amazing potential against possible consequences of this powerful new tool.
